# A Systematic Approach to Diagnostic Laboratory Software Requirements Analysis

**DOI:** 10.3390/bioengineering9040144

**Published:** 2022-03-28

**Authors:** Thomas Krause, Elena Jolkver, Paul Mc Kevitt, Michael Kramer, Matthias Hemmje

**Affiliations:** 1 Faculty of Mathematics and Computer Science, University of Hagen, 58097 Hagen, Germany; elena.jolkver@studium.fernuni-hagen.de; 2 Research Institute for Telecommunication and Cooperation (FTK), 44149 Dortmund, Germany; pmckevitt@ftk.de (P.M.K.); mhemmje@ftk.de (M.H.); 3 ImmBioMed Business Consultants GmbH & Co. KG, 64319 Pfungstadt, Germany; m.kramer@immbiomed.de

**Keywords:** laboratory diagnostics, requirements engineering, gene expression, qPCR, medical diagnostics

## Abstract

Genetics plays an ever-increasing role in medical diagnostics. The requirements for laboratory diagnostics are constantly changing due to new emerging diagnostic procedures, methodologies, devices, and regulatory requirements. Standard software already available for laboratories often cannot keep up with the latest developments or is focused on research rather than process automation. Although the software utilized in diagnostic laboratories is subject to regulatory requirements, there is no well-defined formal procedure for software development. Reference models have been developed to formalize these solutions, but they do not facilitate the initial requirements analysis or the development process itself. A systematic requirements engineering process is however not only essential to ensure the quality of the final product but is also required by regulations such as the European In Vitro Diagnostic Regulation and international standards such as IEC 62304. This paper shows, by example, the systematic requirements analysis of a system for qPCR-based (quantitative polymerase chain reaction) gene expression analysis. Towards this goal, a multi-step research approach was employed, which included literature review, user interviews, and market analysis. Results revealed the complexity of the field with many requirements to be considered for future implementation.

## 1. Introduction

The impact of genetics on medical diagnostics is growing steadily and is already significant. In particular, as part of a so-called “personalized medicine”, genetics enables more accurate diagnoses and targeted treatments [1,2]. One example is the use of metagenomics to determine and categorize the composition of a patient’s gut microbiome, which has been shown to be important for a range of medical fields like human development, diet, immunity, and diseases [3,4]. Another example is the use of gene expression analysis [5] to measure the activity of genes in relation to other genes, which enables diagnostic measures like the upregulation of cytokine-dependent genes, be it under inflammatory conditions or be it in antiviral defense reactions [6].

*Artificial Intelligence for Hospitals, Healthcare & Humanity (AI4H3)* [7] is an R&D white paper developed by FTK (Research Institute for Telecommunication and Cooperation) with associated EU R&D partners in the field of artificial intelligence, big data, and medical analysis. Amongst other features, AI4H3 proposes assistive AI-driven healthcare solutions in the form of a KlinGard open software platform, where decisions must be made to adopt information systems to address their growing demand pressures and support the digital transformation of their facilities and services. KlinGard automatically supports processing of patients’ medical records and medical data for the assistive recognition of salient medical features in various types of medical data including genetic data. Records are also represented in machine-readable and simultaneously semantic formats, supporting enriched, more effective and cost-efficient medical data analysis, medical feature recognition, and medical interpretation. Machine-readable semantic representations enable semantic data fusion, informed medical decision-making support, along with multi-lingual, justifiable, and reproducible medical reporting. This can be applied to, e.g., transparent multi-lingual medical decision explanation support (transparently explaining medical decisions and reports to patients, their families, and clinical staff), and long-term archival support of reproducible medical records for smart healthcare infrastructures. Finally, AI4H3 measures are aimed at building the trust of patients, clinicians, and regulatory authorities in the KlinGard platform.

*OncoADEPT* [8] is another R&D white paper developed by FTK with associated EU R&D partners in the area of personalized medicine with the goal of enabling an existing bioinformatics platform for oncological genetic diagnostic assay development. This improved platform would empower some 7000 labs in the diagnostics and pharma industries and in hospital and research institutions to rapidly and cost-efficiently develop and apply customized molecular genetic assays for cancer treatment.

Medical laboratories have a special role to play in these R&D white papers and other applications, as they bring new research results into clinical practice and hence must constantly evolve. In this context, they must generate, transform, combine, and evaluate data in order to produce individual diagnostic findings.

The volume of data to be processed depends on the application and can be significant. For example, a single complete genome sequencing operation can generate several hundred gigabytes of raw data. Due to the increasing importance of genetics, it is also foreseeable that the total volume of data to be processed will continue to grow at a high rate. This data may also have to be combined with other heterogeneous data in the interests of personalized medicine. Actual data analysis methods are as heterogeneous as the applications, ranging from simple statistical methods to classic machine learning applications and modern methods such as deep learning [9]. From a computer science perspective, diagnostics using genetics is hence a Big Data problem that is usually defined precisely in terms of these three V’s: Volume, Velocity, and Variety [10].

As soon as software is to be deployed in patient management and in particular for diagnostics, it is also usually subject to certain legal regulations or even has to be certified by corresponding bodies. In the European Union, for example, this is stipulated in the IVDR (In Vitro Diagnostics Regulation) [11]. Normative international standards include IEC 62304 [12], which specifies software lifecycle requirements, or ISO 15189 [13], which specifies general requirements for medical laboratories. Not only do these standards specify generic requirements like, e.g., the use of quality management systems, IT security, or usability, they also require that all steps in the software lifecycle and the system design are documented thoroughly. Hence, the software lifecycle does not end with development but includes continuous tasks like PMS (Post-Marketing Surveillance) and PMPF (Post-Marketing Performance Follow-Up).

In summary, challenges arise from the constant change and progress of medicine, technical challenges due to the type and volume of data, and regulatory challenges for use in diagnostics. The recurring challenges and resulting requirements have resulted in the development of reference models. An example in the area of Big Data is the CRISP4BigData model [14]. Applied to the field of laboratory diagnostics and genetics, this model was further developed into the GenDAI model [15], which addresses certain regulatory and medical requirements.

While the aforementioned standards, regulations, and reference models specify requirements that software and the software development process should comply with, they do not recommend or require a specific approach or methodology for the software development process. Hence, they also don’t provide any guidance on how to obtain and analyze user requirements. This problem is addressed here in this paper. The research question we focus on here is: How can we systematically analyze user requirements for laboratory software from such multi-dimensional sources and document them in a standard-compliant way?

As a starting point, the research method of Nunamaker et al. [16], which covers a general approach to systems development, can be utilized. The method describes four strategies — *Observation*, *Theory Building*, *Systems Development*, and *Experimentation* — which can be alternated as required. In the context of requirements analysis, the first two strategies are particularly relevant.

Applied to our research question, the following research goals form the basis of our structured research and development approach: analyze the state of the art (SotA) in science (Observation, RG-O1), analyze applicable standards, and regulations (Observation, RG-O2), analyze the state of the art in technology (Observation, RG-O3), analyze laboratory practice (Observation, RG-O4), analyze existing solutions (Observation, RG-O5), derive remaining requirements (Observation, RG-O6), create a conceptual model of the solution (Theory Building, RG-T1), create prototypical proof-of-concept implementation (Systems Development, RG-D1), evaluate conceptual model (Experimentation, RG-E1), and evaluate prototypical proof-of-concept implementation (Experimentation, RG-E2).

The remainder of the paper will discuss this structured research and development approach and the results obtained using as a real-life example a small laboratory based in Germany and the application area of gene expression analysis. The laboratory wants to optimize its analysis processes, stay up to date with current research, and ensure compliance with future regulations. Section 2 discusses the methods used in the requirements analysis. The results of this analysis are discussed in Section 3 and conclusion and future work in Section 4.

## 2. Methods

**Figure 1:**
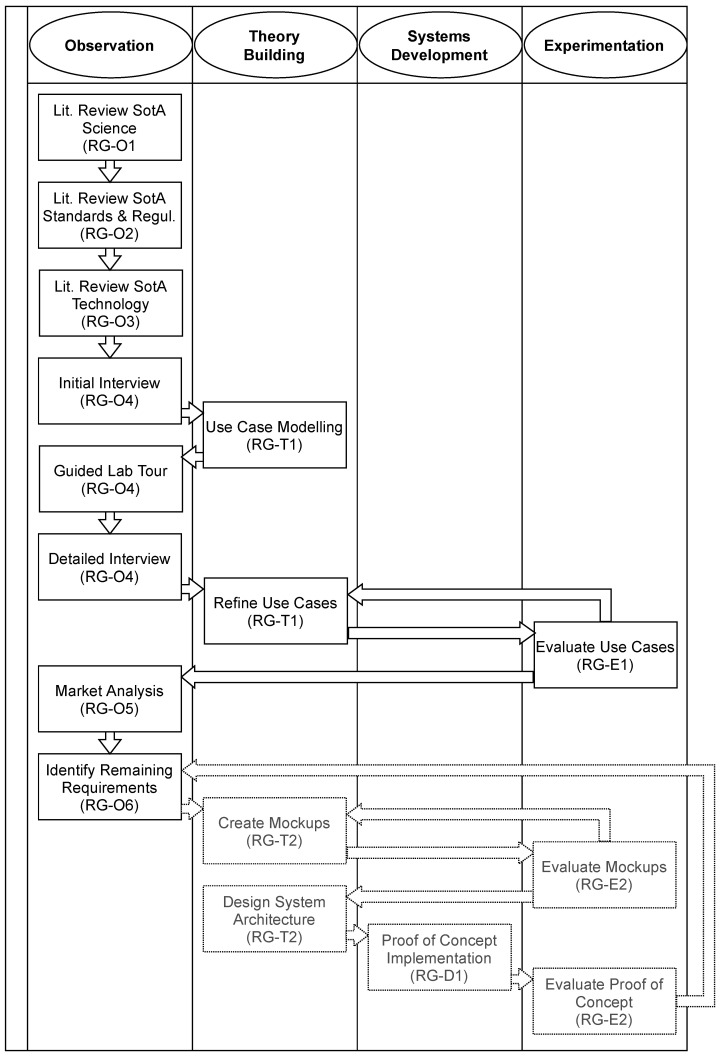
Research approach in the framework of Nunamaker et al. [16].

The systematic process depicting our research goals in the framework of Nunamaker et al. [16] is shown in Figure 1. The Figure 1 arrows indicate the sequence of activities, not necessarily a dependency. Columns show the overall research strategy employed from Nunamaker et al. [16]. The concrete research goal that the activity targets, is given in parenthesis. Some research goals are targeted by more than one activity. Dashed activities have not been executed yet and are the subject of ongoing future work. To reach the described observation goals, we followed the User-Centered System Design process developed by Norman and Draper [17] and applied such methods as literature review, market research, interviews, or actually watching users at work to target our observational research goals.

To understand the state of the art in science (RG-O1), the first activity was a well-scoped literature review on the topical areas of RT-qPCR (reverse transcription quantitative polymerase chain reaction) technology and gene expression analysis methods using well-established scientific publication search engines. This quickly led to a review of the relevant standards and regulations in the field (RG-O2), including state-of-the-art technology (RG-O3). This order enabled us to account for regulatory requirements whilst reviewing the technology, which is pivotal in the tightly regulated field of laboratory analyses. Relevant literature for the latter goals was identified using a combination of keyword searches and cross-references from other relevant literature.

Based on the results of the literature review, an initial interview was then conducted in writing with the head of the laboratory (RG-O4). Questions were chosen to understand the scope of activities in the laboratory, to compare the results of the literature review with laboratory practice, and to understand practical requirements. This includes questions about the type of analyses performed, the hardware and software used, initial questions about the sequence of analyses, and quality control measures. Examples of these questions are: (1) What hardware and software do you currently use for gene expression analysis?, (2) How many samples/genes do you process currently at the same time?, (3) How many do you plan to process in the future?, (4) What kind of analysis do you perform on the quantification data?, (5) How do you evaluate the statistical significance of results?, and, (6) What kind of meta-analysis, spanning multiple runs, do you regularly conduct?

Utilizing the answers to the questionnaire and the initial literature research a *Preliminary Conceptual Model* was created that describes the existing laboratory process in the form of use cases and flow charts (RG-T1). For this purpose, the process was first divided into different phases and then for each phase, the user stereotypes involved, as well as associated use cases were described in UML (Unified Modelling Language). To limit the research scope, only the use cases most relevant to the laboratory were selected. These were identified based on the written interview results.

Besides modeling the existing process, a possible target model was also drafted that strives for the highest possible degree of automation while simultaneously reducing sources of error. In doing so, it was consciously accepted that this target image could possibly be naive or even erroneous due to a lack of detailed understanding. However, it is precisely this “naivety” that appeared desirable in this initial modeling, as it allows existing processes to be rethought and questioned. If instead, modeling only takes place towards the end, i.e., after the existing process has been understood in detail, there is a risk that existing processes will simply be adopted and no longer questioned.

The detailed analysis of the existing process for optimization potential can be understood as a market-pull strategy, while the independent modeling of an optimal target image is considered a technology-push strategy. Hence, in this sense, a combined push-pull strategy was applied.

**Figure 2:**
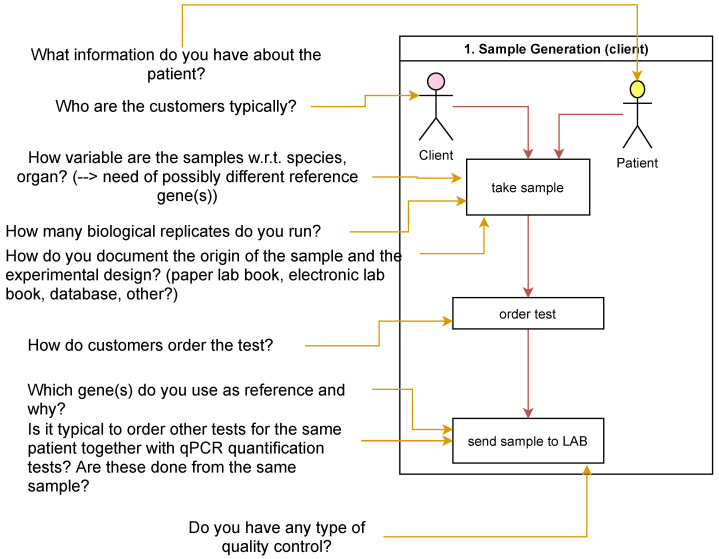
Example questions for the laboratory use case of sample generation within the laboratory.

Detailed questions were then formulated for each known and imagined future use case (Figure 2) in the *Preliminary Conceptual Model*. The questions were designed to gain a better understanding of the process itself and to identify optimization potential and possible sources of error in order to obtain starting points for future improvements (RG-O4).

The drafts and the associated detailed questions were presented to the laboratory. Corrections and comments on the models and the answers to the questions were documented in the form of a transcribed interview. The interview was accompanied by an on-site visit at the laboratory, during which all workstations were inspected. For this purpose, the path of an exemplary sample from the initial order to the final findings was followed. This made it possible to uncover further work steps that might have been lost through the transcribed interview alone (RG-O4). This applies in particular to manual steps with potential for optimization.

Based on the interview and the site visit, the *Preliminary Conceptual Model* was revised again to incorporate new findings (RG-T1). The refined use cases along with clarifying questions were presented to the lab in a feedback loop (RG-E1) until a consensus for a *Conceptual Model* was reached.

Subsequently, a market analysis was conducted to check which software can support one or more of the current and future use cases (RG-O5). For this purpose, a *qPCR Software Feature Overview* list was created using the list from Pabinger et al. [18] supplemented with additional tools, which were either out-of-scope for the original publication or were released afterward. Additional features for evaluation were also added to the list where previous research activities indicated that they are relevant for the laboratory or otherwise useful. To check the availability of a feature, publicly available information was used. This included data on the manufacturer’s websites, user manuals, screenshots, or published papers. In some instances, the software was installed to further validate the features.

Functionalities that are not covered at all or only insufficiently by current software were identified and documented (RG-O6). Software that showed the most promising evaluation was selected for a more thorough analysis, which was documented in a *Coverage Matrix of Prioritized RT-qPCR Analysis Process Steps*. This matrix was created in several steps. First, the general process steps and associated software features within the qPCR analysis process were assigned a priority. Features already in use were assigned priority 1 (must have), features already planned were assigned priority 2 (should have), possible future features were assigned priority 3 (could have), and features not relevant for the example laboratory in the foreseeable future were assigned priority 4 (not relevant). Finally, the existence of the feature in the software evaluated was checked. The goal of assigning a priority was to facilitate a weighted comparison that is not based purely on the feature count. Using the same reasoning, an additional comparison was conducted using overall feature areas and a list of specific functionalities offered by the software products in this feature area (*Detailed qPCR Software Feature Comparison*).

The creation of mockups and a system architecture (RG-T2) based on the documented requirements, prototypical implementation (RG-D1), and evaluation (RG-E2) were considered as out-of-scope for this initial analysis.

## 3. Results

Analysis of the state of the art in science revealed the complexity of the field with numerous techniques and continuous scientific advancement. Examples include the type of analysis performed (relative quantification vs. absolute quantification) [19] or techniques to improve the accuracy of gene expression quantification [20,21]. As a result, the system architecture for a proposed solution needs to be easily configurable and extensible to accommodate future changes.

Similar complexity was found in the analysis of relevant standards and regulations. In addition to international official standards like ISO 15189 [13] and IEC 62304 [12], there are also unofficial standards like the MIQE (minimum information for publication of quantitative real-time PCR experiments) guidelines for qPCR experiments. Although MIQE is primarily a publication standard, it defines common terminology and hence is also employed by some software tools [22]. Lastly, as the laboratory is located inside the European Union, local regulations like IVDR apply [11].

The IVDR, as an example, is comprised of over 200 pages with cross-references to other regulations. To navigate this complexity, some organizations offer guidance and consulting to help laboratories, device manufacturers, or software developers comply with the regulation terms [23]. Similar offerings exist for other standards and regulations.

**Figure 3:**
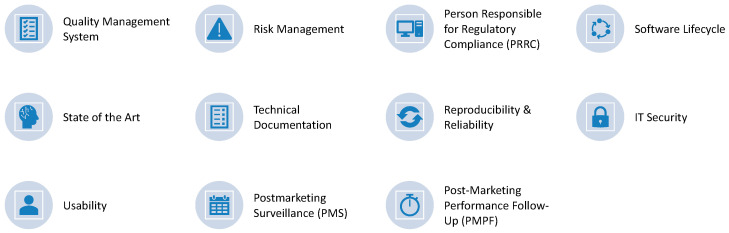
Relevant requirements from IVDR (In Vitro Diagnostics Regulation) and related regulation.

Concrete requirements resulting from the aforementioned standards and regulations for software development in the laboratory context include, for example, using current state of the art technology, taking into account the complete software lifecycle, and IT security. Several more technical and administrative measures are shown in Figure 3 and have also been described in Krause et al. [5].

In the area of technology, the reference models introduced in Section 1 can potentially be used to evaluate existing solutions or to develop new solutions. The GenDAI model was developed in parallel to this prestudy and hence already contains some of the insights obtained here as well as insights from other medical fields. The investigation into technology also revealed the RDML (real-time PCR data markup language) data format as a potential standard for qPCR experiments [24].

**Figure 4:**
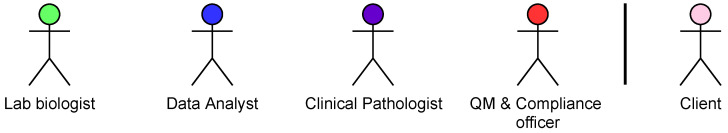
Identified user stereotypes.

The initial interview facilitated identifying the steps usually taken during the exploratory assay development phase as well as the steps of a routine assay. This classification facilitated limiting the scope of the investigation to use cases being important for the routine assay and to identify user stereotypes for use in the *Preliminary Conceptual Model*. The principal user stereotypes that have been identified are the *Lab Biologist*, the *Data Analyst*, the *Clinical Pathologist*, a *QM & Compliance Officer*, and the *Client* (Figure 4).

**Figure 5:**
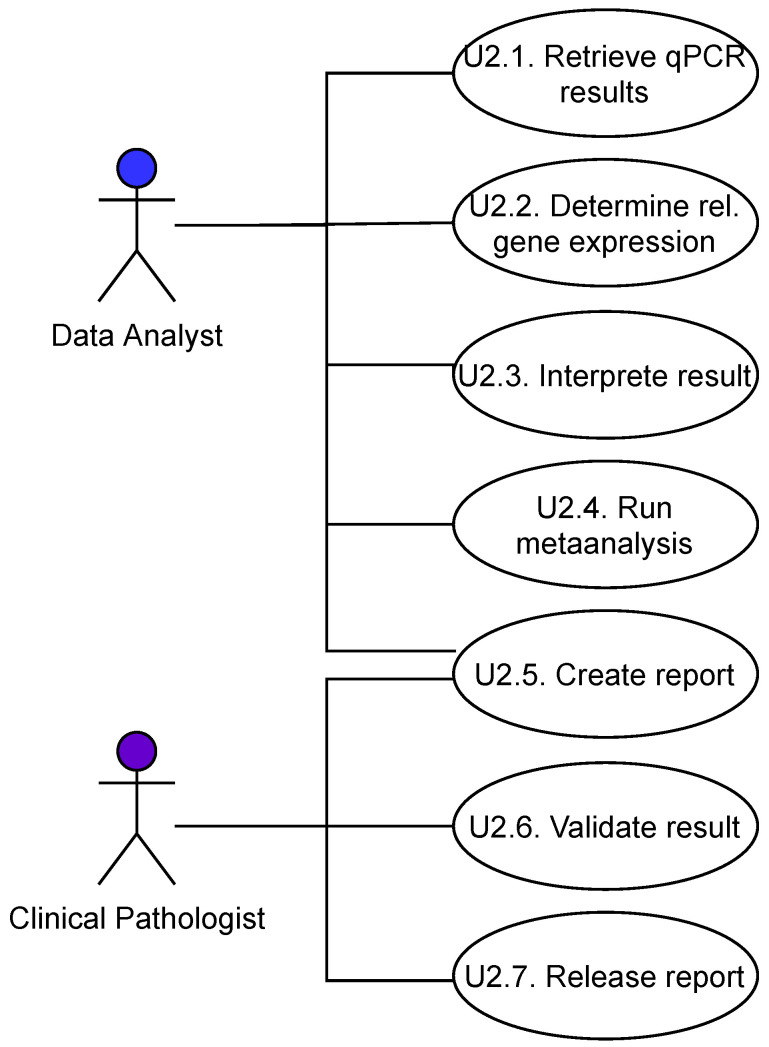
Example use cases.

For each of these user stereotypes, several use cases have been identified that are relevant for the scope of the *Preliminary Conceptual Model*. Some examples of these use cases are shown in Figure 5. The use cases are described further with flow charts and textual descriptions.

The guided lab tour, together with the in-person interview, proved effective in understanding the needs and limitations of the current process and refining the *Preliminary Conceptual Model* into the final *Conceptual Model*. One key insight is that the laboratory currently employs several manual steps that suggest potential candidates for optimization. This can at least be partially attributed to the use of several different software products that provide little support for automatization and utilize several different incompatible data formats making integration between products difficult.

Market analysis focusing on available software and its core features yielded the *qPCR Software Feature Overview* list shown in Table 1 with several tools previously described in Pabinger et al. [18], as well as additional tools which were either out-of-scope for the original paper or not available then, namely ExpressionSuite Software [25], Factor-qPCR [26], GenEx [27], geNorm [20], PIPE-T [28], Q-Gene [29], qbase+ [21,22], RealTime StatMiner [30], and SoFAR [31]. According to the *Preliminary Conceptual Model*, the feature list was adapted to lab requirements and extended to the tool’s main purpose, data import capability, supported data formats, melt curve analysis support, selection of reference genes, OS/Framework, last update, and costs.

The tools with the largest range of features obtaining updates within recent years are the commercial tools, qbase+ [22] and GenEx. These tools were analyzed and compared in more detail. Table 2 shows the results of this *Coverage Matrix of Prioritized RT-qPCR Analysis Process Steps* for various features and processing steps together with the importance of features for each stereotype. For each relevant process step, the priority for individual user stereotypes ranging from priority 1 (must have) to priority 4 (not relevant) is given. The last two columns indicate the availability in qbase+ and GenEx respectively.

Overall, both tools cover similar features. To understand the differences between the tools better, a *Detailed qPCR Software Feature Comparison* for the features available in qbase+ and GenEx was completed to supplement the previous list. For a set of generic feature areas, the qualitative comparison in Table 3 shows the concrete manifestation and scope of support for both products. Features found in both tools span both columns. Features, which were reported in either documentation and could not be mapped to a description in the other documentation, are given in the individual tool’s column.

Both tools cover a large variety of analysis steps, yet set a focus on different parts of the analysis. In particular, GenEx stands out in respect of its data analysis features, providing multiple statistical methods, visualizations, support for experimental design, as well as predictive tools. Furthermore, GenEx documents the pre-processing steps, which have been performed by the user. On the other hand, the focus of qbase+ lies in pre-processing and QC steps. It offers improved control of sample normalization and scaling as well as quality checks of different sample types, such as technical replicates, negative controls, reference gene stability, and PCR specificity. Furthermore, it enables general connectivity with a multitude of qPCR cyclers due to compatibility with the RDML format.

qbase+ and GenEx offer extensive features for performing gene expression analysis by qPCR. A downside of both tools is that they are optimized for use in research rather than for regular use in medical laboratories. This is evident from the user interface that treats analyses as part of a specific project rather than providing features for automated processing of many samples. Consequently, the main application area of the analyzed software is the phase of the assay development, whereas the multitude of required user interactions poses a hindrance for automated routine operation.

## 4. Conclusions and Future Work

The requirements for laboratory software are high-spec and stem from various areas, such as information technology, the state of the art in science, regulations, and standards. In addition, these requirements are constantly changing, which causes a continuous need for adaptation within laboratories. The selection of new software or the development of in-house solutions to meet this need for adaptation is complex due to the many requirements. A systematic approach to identifying and analyzing these requirements is not only useful but can also address regulatory requirements for documentation.

One possible methodology has been outlined in this paper. Based on the strategies of Nunamaker et al. [16] and methods like User-Centered System Design [17], the requirements analysis was demonstrated on the concrete example of gene expression analysis in a small laboratory.

The requirements analysis facilitated classification and evaluation of existing software products. It was also possible to identify gaps that have not yet been covered by existing tools. Keeping the client laboratory in a tight communication loop and adopting their feedback provided rapid adaptation to their needs and eventually confidence in accepting the results, which can be regarded as an indication of the validity of the methodology.

We believe that the methodology shown here can serve as a template for requirements analyses of software in laboratory diagnostics. In the future, we plan to extend the methodology to the architecture, design, development, and evaluation of software in laboratory diagnostics. One of the remaining challenges within this work will be to address changing requirements stemming from scientific progress and regulations.

## Figures and Tables

**Table 1:** qPCR Software Feature Overview. “+” symbolizes presence of feature, “−” absence of its reference in documentation, “nd”: not determined.

Tool	Main Purpose	Data Import	Data Format	PCR Efficiency Estimation	Melt Curve Analysis	Selection of Reference Genes	Calculates Cq from Raw	Error Propagation	Normalization	Absolute Quantification	Relative Quantification	Outlier Detection	NA Handling	Statistics	Graphs	MIQE	OS/Framework	Last Update	Costs	Reference	Count “+”
CAmpER	Quantification	Raw	FLO, ABT, CSV, REX, TXT	+	nd	nd	+	−	−	−	+	nd	−	−	+	−	Web Service	2009	discontinued	[18]	4
Cy0 Method	Quantification	Raw	XLS, TXT, DOC	−	−	−	+	−	−	−	−	−	−	−	−	+	Web Service	2010	free	[18]	2
DART-PCR	Quantification	Raw	XLS	−	−	−	+	−	+	−	+	+	−	−	+	−	Windows, Excel	2002	free	[18]	5
Deconvolution	Quantification	Raw	TXT	−	−	−	−	−	−	+	−	−	−	−	−	+	Perl based	2010	free	[18]	2
ExpressionSuite Software	Quantification	Raw	EDS, SDS	−	+	−	+	−	+	−	+	+	−	+	+	+	Windows	2019	free	[25]	8
Factor-qPCR	Inter-Run Calibration	Raw, Cq	XLS, RDML	−	−	−	−	−	+	−	−	−	−	−	−	+	Windows, Excel	2020	free	[26]	2
GenEx	Quantification	Cq	TXT	+	−	+	−	−	+	+	+	+	+	+	+	+	Windows	2019	commercial	[27]	10
geNorm	Reference Gene Selection	see qbase+	see qbase+	−	−	+	−	−	−	−	−	−	−	−	−	−	see qbase+	2018	free	[20]	1
LinRegPCR	Quantification	Raw	XLS, RDML	+	−	−	+	−	−	+	−	+	−	−	+	+	Windows	2021	free	[18]	6
LRE Analysis	Quantification	Raw	XLS	−	−	−	−	−	−	+	−	−	−	−	−	+	MATLAB based	2012	free	[18]	2
LRE Analyzer	Quantification	Raw	XLS	−	−	−	−	−	−	+	−	−	−	−	+	+	Java based	2014	free	[18]	3
MAKERGAUL	Quantification	Raw	CSV	−	−	−	+	−	−	+	−	−	−	−	−	+	Server-Client Arch.	2013	free	[18]	3
PCR-Miner	Quantification	Raw	TXT	+	−	−	+	−	−	−	−	−	−	−	−	+	Web Service	2011	free	[18]	3
PIPE-T	Quantification	Cq	TXT	−	−	−	−	−	+	+	+	+	+	+	+	−	Galaxy	2019	free	[28]	7
pyQPCR	Quantification	Cq	TXT, CSV	+	−	−	−	+	+	−	+	−	+	−	+	+	Python based	2012	free	[18]	7
Q-Gene	Experiment Design and Analysis	Cq	XLS	+	−	−	−	−	+	−	+	−	−	−	+	−	Windows, Excel	2002	free	[29]	4
qBase	Quantification	Cq	XLS, RDML	+	−	+	−	+	+	−	+	+	−	+	+	+	Windows, Excel	2007	discontinued	[18]	9
qbase+	Quantification	Cq	XLS, RDML	+	−	+	−	+	+	+	+	+	−	+	+	+	Windows, Mac	2017	commercial	[22]	10
qCalculator	Quantification	Cq	XLS	+	−	−	−	−	+	−	+	−	+	−	+	−	Windows, Excel	2004	free	[18]	5
QPCR	Quantification	Raw	CSV, RDML	+	−	−	+	+	+	−	+	−	+	+	+	+	Linux Server	2013	free	[18]	9
qPCR-DAMS	Quantification	Cq	XLS	−	−	−	−	−	+	+	+	−	+	−	−	+	Windows	2006	free	[18]	5
RealTime StatMiner	Quantification	Raw, Cq	TXT	−	−	+	−	+	+	−	+	+	+	+	+	+	Windows	2014	commercial	[30]	9
REST	Quantification	Cq	TXT	−	−	−	−	+	+	−	+	−	−	+	+	+	Windows	2009	free	[18]	6
SARS	Quantification	Cq	XLS, TXT	−	nd	nd	−	−	+	−	+	nd	−	+	−	+	Windows	2011	discontinued	[18]	4
SoFAR	Automated Quantification	Raw	ABT + FLO	+	+	−	+	−	−	−	−	−	−	−	+	−	Windows	2003	discontinued	[31]	4

**Table 2:** Coverage matrix of prioritized RT-qPCR analysis process steps for qbase+ and GenEx with assigned priority for individual user stereotypes.

Process Step	Description	User Stereotype							Commercial Software	
		**Method Validation**	**Order Entry**	**Cycler**	**Lab Biologist**	**Data Analyst**	**Clinical Pathologist**	**Compliance Manager**	**GenEx**	**qbase+**
Import of Experiment Metadata and Data Storage	Import of sample information		1						n.a	n.a
Experiment Design	(Fractional) factorial design when testing for multiple impact factors	3	4						+	−
Power Analysis	Estimate required number of biological replicates to determine statistical difference between groups	3	4						+	−
Data Import	Transfer of data from cycler to analysis workflow				1				Cq	Raw, Cq
Data Format	Format of the imported data				1				TXT	XLS, RDML
Cycler Compatibility	System accepts data from cycler used by laboratory				1				−	+ (as RDML)
PCR Efficiency Estimation	For correct estimation of target initial concentration	1			3				+	+
Selection of Reference Genes	Check expression stability of candidate reference genes	1			2				+	+
Sample QC (documentation)	RNA integrity and purity, DNA absence				1				n.a	n.a
Cq Calculation	Determine Cq from fluorescence data			1					−	−
Error Propagation	Propagating of measurement uncertainty through functions based on the measurement’s value				3				−	+
Normalization	Inter-Run Calibration across devices or experiments				2				+	+
Relative Quantification	Determine fold change values based on a reference				1				+	+
Absolute Quantification	Calculate absolute quantification values				4				+	+
Outlier Detection	Calculate fold change values after relative quantification				3				+	+
NA Handling	Remove NA automatically or impute missing values				3				+	−
Statistical Tests to assess Differential Gene Expression	Perform appropriate statistical test to determine statistical differences between groups					4			+	+
Reporting (Graphs)	Create graphs					1			+	+
Reporting (Interpretation)	Interprete results and write coherent report					1	1		n.a	n.a
MIQE	Store MIQE-relevant information							1	+	+
Automatization	Automate analysis workflow					2			−	−

**Table 3:** Detailed qPCR software feature comparison of qbase+ vs. GenEx with individual features within an area mapped to one or both tools.

Feature Area	GenEx	qbase+
Experimental Design	Sample number	
	Experimental design optimization	
Pre-processing of Data	Logged in a file	Inter-run calibration
	Interplate calibration	
	PCR efficiency correction, estimation from standard curve	
	Normalize to sample amount (volume processed, amount of RNA used for reverse transcription, or cell count)	
	Normalize to reference genes/samples	
	Normalize to spike	Normalize to global mean
	Missing data handling (detection and interpolation)	Normalize to Global mean on common targets
	Convert to log scale	Scaling to mean, max, min, sample, group, positive control
	Cq averaging	
	Relative quantities and fold changes	
Quality Control	Correct for genomic DNA background	User-defined quality thresholds
	Average technical replicates	Technical replicates (Replicate variablity)
	Primer Dimer Correction	Pos. and neg. controls (Cq boundaries)
		Stability of reference targets
		Sample specific characteristics (M value, coefficient of variation)
Finding optimal reference genes	geNorm	
	NormFinder	
	Geometric averaging	
Absolute Quantification	Standard curves	
	Reverse Regression	
	Limit of detection (LOD) estimation	Copy number analysis
Correlation	Spearman rank correlation coefficient	
	Pearson correlation coefficient	
Statistics	Descriptive statistics	
	False Discovery Rate Correction	
	Student’s t-test paired, unpaired	
	Non-parametric tests (Mann-Whitney, Wilcoxon signed rank)	
	One-way ANOVA	
	Two-way ANOVA	
	Nested ANOVA	
	Trilinear decomposition	Survival analysis (Cox prop. hazards)
Cluster Analysis	PCA	
	P-curve	
	Hierarchical clustering/dendogram	
	Heatmap analysis	
Sample Classification	Self-organizing map (SOM)	
	Artificial neural networks (ANN)	
	Support vector machine (SVM)	
Concentration Prediction	Partial least square (PLS)	
Plots	Correlation Plot/Scatterplot	
	Bar plots	
	Line plots	
	Box and whiskers plot	
	Heatmap	

## Data Availability

Not applicable.
